# Childhood poisoning in Warri, Niger Delta, Nigeria: A ten year retrospective study 

**DOI:** 10.4102/phcfm.v4i1.321

**Published:** 2012-06-22

**Authors:** Gilbert I.M. Ugwu, Blessing O. Okperi, Eunice N. Ugwu, Nekwu E. Okolugbo

**Affiliations:** 1 Department of Paediatrics Delta State University, Teaching Hospital Oghara, Nigeria; 2 Chevron Hospital Warri Nigeria; 3 Department of Otorhinolaryngology, Delta State, University Teaching Hospital Oghara, Nigeria

## Abstract

**Background:**

Childhood poisoning is a common but avoidable problem in developing countries such as Nigeria.

**Objectives:**

To determine the pattern of childhood poisoning in the Warri Niger Delta Region of Nigeria.

**Method:**

The case notes of all the children seen at Central Hospital Warri, the reference base for the region and GN Children Clinic the pioneer children’s hospital in the region. This is a review of cases over a ten year period, from 2000 to 2009. The information obtained was analysed.

**Results:**

A total of 156 children aged 0–16 years diagnosed with poisoning were seen at the central hospital in Warri and at the GN Children’s Clinic which is also in Warri over a 10 year period from 2000 to 2009 under review. The male to female ratio is 2:1, and 75% of the children were aged 5 years or less. Most of the patients were from the low socio-economic class. Most of the poisoning was unintentional and occurred through ingestion (97.6%). Kerosene was the major substance leading to poisoning (56.6%). Alcohol ranked second in the study. Poisoning from drugs was the third most common source of poisoning and in that category most of the indices were in the highest income group. Most of the patients presented with mild symptoms and the mortality rate was 7%.

**Conclusion:**

Kerosine was found to be the most common source of poisoning. Most of the poisoning was unintensional and deaths cause by this form of poisoning can be prevented with proper health education and effective enactment of laws that will reduce the incidence of childhood poising.

## Abstrait

L’empoisonnement des enfants à Warri, Delta du Niger, Nigeria : Une étude rétrospective sur 10 ans

**Présentation:**

L’empoisonnement des enfants est un problème courant mais évitable dans des pays en développement tels que le Nigeria.

**Objectifs:**

Déterminer la tendance de l’empoisonnement des enfants à Warri, dans la région du Delta du Niger au Nigeria.

**Méthode:**

Les notes de cas de tous les enfants vus à l’hôpital central de Warri, la base de référence pour la région et l’Hôpital pour enfants GN, le premier hôpital pour enfants dans la région. Il s’agit d’une étude de cas sur une période de dix ans, de 2000 à 2009. Les informations obtenues ont été analysées.

**Résultats:**

Au total, 156 enfants âgés 0–16 ans diagnostiqués avec un empoisonnement ont été vus à l’hôpital central de Warri et à l’hôpital pour enfants GN, qui se trouve également à Warri, sur une période de dix ans allant de 2000 à 2009. La proportion garçons/filles est de 2/1, et 75% des enfants étaient âgés de 5 ans ou moins. La plupart des patients appartenaient à la classe socio-économique inférieure. La plupart des empoisonnements étaient non intentionnels et se sont produits par ingestion (97.6%). Le kérosène était la principale substance responsable des empoisonnements (56.6%). L’alcool arrivait en seconde position dans cette étude. L’empoisonnement par des médicaments était le troisième facteur d’empoisonnement le plus courant et, dans cette catégorie, la plupart des indices appartenaient aux groupe de revenus les plus élevés. La plupart des patients souffraient de légers symptômes et le taux de mortalité était de 7%.

**Conclusion:**

Le kérosène était la source d’empoisonnement la plus courante. La plupart des empoisonnements étaient non intentionnels et les décès provoqués par cette forme d’empoisonnement peuvent être évités grâce à une éducation sanitaire adéquate et une promulgation effective des lois permettant de réduire l’incidence de l’empoisonnement d’enfants.

## Introduction

Poisoning is a major cause of morbidity and mortality amongst children^1,2^ and it is the third major killer in the United States of America (USA).^3^ Though accurate data are difficult to come by in developing countriess such as Nigeria, it is estimated to be the fourth major cause of ill health and death in developing countries.^4^ The most common poisons vary according to age, country, and whether the country is classified as ‘developed’ or ‘developing’.^3^ Contrary to the situation in developed countries, kerosene is a source of poisoning in developing countries as a result of inadequate control of access to the substance.^4^ Drugs are another source of poisoning and the control of the production of these drugs is usually grossly inadequate. Recently, a teething mixture called ‘my pickin’ caused acute renal failure in children in Nigeria.^5^ Poisoning as a result from ingesting the substance was caused by the presence of ethylene glycol in place of propylene glycol in the preparation of paracetamol, which is a major component of the mixture. There have also been reported cases of death of children due to the wrong compounding of paracetamol in Nigeria^6^. 

Exposure to products such as kerosene is more common in developing countries where this is used as fuel for cooking, making it easily accessible to children.^7^ Moreover, kerosene is a colourless liquid and usually stored in containers similar to those used for storing water. Caustic soda, which is used for local soap production, is another cause of childhood poisoning in Nigeria, accounting for 20% of childhood poisoning as seen at the Obafemi Awolowo University in Ile-Ife, Nigeria.^8^ Very recently in July 2010, there has been a report on the high incidence of lead poisoning amongst children in northern Nigeria, especially in Zamfara State. This is as a result of mining activity in search of gold and this has drawn international attention, as it is causing a significant increase in the mortality rate.^9^ The purpose of this study was to determine the pattern of childhood poisoning in Warri in the Niger Delta region of Nigeria, and the increase in the mortality rate as a result of it. This will help in the efforts aimed at reducing childhood mortality.

## Methods and materials

One hundred and fifty six cases of poisoning amongst children were reported and the offending substances were identified as seen at the Central Hospital Warri (a major referral public hospital) and at GN Children’s Clinic (one of the four major private children’s hospitals in the area). These hospitals serve more than three Local Government Areas (LGAs) with a population of 2.6 million people according to the 2006 census held in Nigeria^10^; the retrospective review was held from January 2000 to December 2009.

The case notes of children with symptoms indicating poisoning at the Central Hospital Warri and GN Children’s Clinic were retrieved and analysed. Information obtained include:

- their age
- their sex
- the offending substance
- the mode and route of poisoning (i.e. ingestion or otherwise)
- whether the poisoning was unintentional or deliberate 
- where the source of poisoning or its container was found after poisoning (i.e. whether still found at the original place of storage)
- the quantity consumed 
- the presenting symptom(s)
- the circumstances surrounding the poisoning
- home management before presentation (if any) 
- the severity of poisoning and the time lapse between poisoning and presentation
- the patients’ place of domicile
- the occupation of the parents 
- the level of education of parents and patients where necessary 
- the treatment given at the hospitals and the outcome in terms of recovery (i.e. full or partial recovery, complications, mortality, etc.)
- follow-up records

## Results

A total of one hundred and fifty six children were seen in these hospitals during the period from 2000 to 2009. All the cases where children were poisoned were as a result from ingesting poisionous substances. Of these cases, 104 patients were male children, and 52 were female children giving a male: female ratio of 2:1; the yearly distribution is shown in Table 1.

**TABLE 1:**
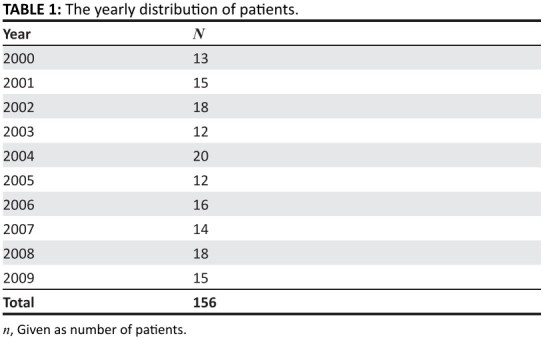
The yearly distribution of the parents.

**FIGURE 1:**
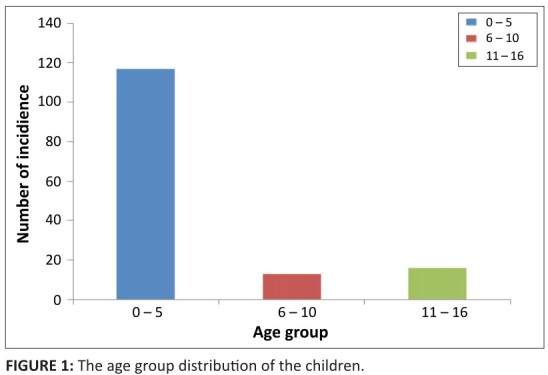
The age group distribution of the children.

Table 1 shows that the incidence of poisoning per annum was virtually uniform except for 2002, 2004 and 2008 where there were appreciable increases. One hundred and seventeen (117) children were aged 5 years and less, representing 75% of the children; 8.5% of the children who exhibited symptoms associated with poisoning were in the school age bracket (> 5–10 years), and 16 children or approximately 16.5% were aged > 10–16 years (Figure 1).

**FIGURE 2:**
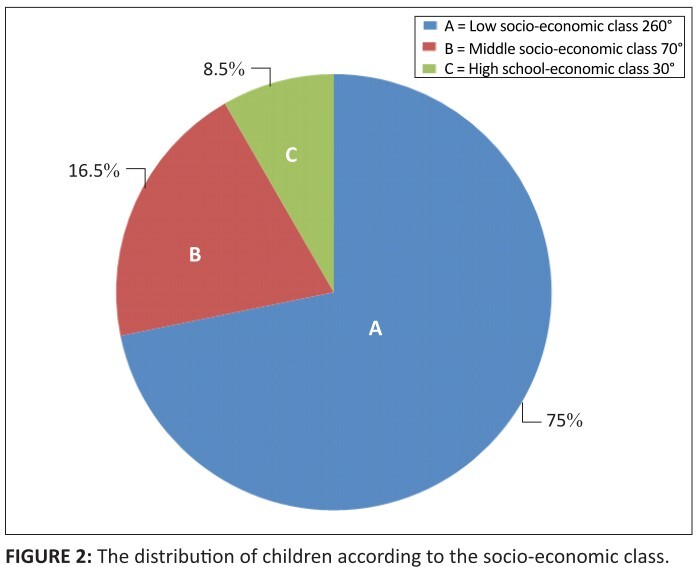
The distribution of children according to the socio-economic class.

Figure 2 represents the socio-economic class of the children involved in the study. One hundred and twelve of the children were from the low socio-economic class whilst 31 of the children were from the middle class and 13 children from the high income group; 97.6% (152) of the poisoning cases was unintentional whilst 2.4% (or 4) were intentional. Kerosene was responsible for poisoning in 88 of the patients representing 56.6%. The second most common source of poisoning is alcohol, which accounts for 19 of the cases (12.3%); 16 of the cases (10.5%) resulted from poisoning from drugs. The major drugs were benzodiazepines, methyldopa, ferrous sulphate, metformine, and cypron (cyclorheptadine). Of interest is that more children in the middle socio-economic class were poisoned from drugs (8 out of 16, representing 50%). This was followed by those in the higher socio-economic class (6 out of 16, representing 37.5%). Only 2 children (12.5%) were from the low socio-economic stratum. Only about 38.2% of the involved drugs were found at the place of storage after the poisoning episode. Caustic soda accounted for 8.4% or 13 cases of childhood poisoning in Warri.

The intentional poisoning cases involved children in the > 10–16 years age bracket. Three of these children were female and one was male. The cause of the intention in two of the cases involving female children was due to boyfriend–girlfriend problems. In one case, a child overdosed on her father’s glibenclamide (anti-diabetic drugs) whilst the other child used diazepam. The other two children with intentional poisoning (i.e. the boy and the third girl) were caused by an alcohol overdose.

Table 2 shows that 6% of the causes of poisoning were due to administering native concoctions; 2% of the causes of poisoning in children resulted from crude oil. Other offending agents, namely methylated spirit, nail polish and mushrooms, combined were responsible for 4.2% of the children. Table 2 represents these findings; 70.4% of the patients presented as mild cases whilst 15.6% had moderate poisoning and 14% were severe, as shown in Table 3.

**TABLE 2:**
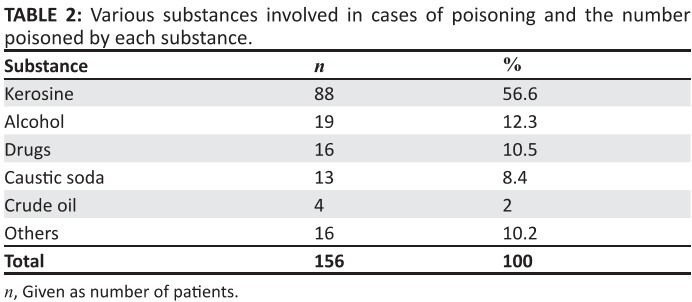
Various substances involved in cases of poisoning and the number poisoned by each substance.

**TABLE 3:**
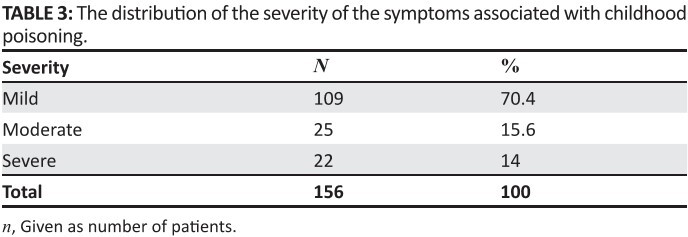
The distribution of the severity of the symptoms associated with childhood poisoning.

Table 3 reveals that 8.6% of the patients had complications. The complications included aspiration pneumonitis, oesophagitis, recurrent pneumonia, and seizures. Palm oil was given orally as an antidote to poisoning in 76.1% of the children. Other antidotes given include palm kernel oil and olive oil, especially the oils blessed by the pastor.

In terms of the mortality rate 7% of the children died. Of these children who had passed away 72.7% of the deaths were due to consuming native concoctions, one from kerosene, drug and caustic soda and nail polish (i.e. 9% each). One of the four children who intentionally poisoned themselves died; 63.6% of these children were from the low socio-economic class, 27.3% were from the middle class, and 9.1% were from the high income class.

## Discussion

Poisoning in childhood is a common phenomenon worldwide and this is because of the innovative, inquisitive nature of children and their mouthing tendency.^11,12^ A total of 156 cases involving children were recorded in our study, which was more than the number seen in Ile-Ife over the same ten year period, but much less than seen in India.^13^ In the Indian study, out of 2720 calls received by the National Poison Information Centre over a three year period, 995 calls involved children, which is much higher than the finding in our study. In another study in India conducted over a two year period, 111 children were examined,^14^ compared with 156 children who were examined over a ten year period in our study. All the patients were poisoned from ingestion and this is similar to the second study in India where almost all of the children (96.9%) were poisoned orally.^14^ The incidence of childhood poisoning was more common in male children than in female children, which is the same as experienced in Ile-Ife and worldwide as well^8,13,15,16^. In fact, a male : female ratio of 2:1 was approximately what was obtained in India.

 Over the ten year period of review, there were peaks in 2002, 2004 and 2008. These correlated with periods of kerosene or fuel scarcity in Warri. More than three quarters of the patients were 5 years and less. Whilst 80.95% of the cases in Ile-Ife were less than 5 years in age, our study showed that 75.6% of the patients were less than 5 years. In Spain, the incidence in children 4 years and younger was 67%17, whilst a study in Babol, Iran showed that 81% of those involved in childhood poisoning were below 5 years^18^. The lower figures in our study compared to the study in Ile-Ife may be due to exposure to more substances in our study than in the Obafemi Awolowo University Ile-Ife study^8^. In that study, over 90% of the patients were exposed to only kerosene, caustic soda and traditional medications as opposed to more than eight items in our study. The incidence of poisoning as noted in our study was higher in the adolescent age group (11–16 years) than the school age group bracket (6–10 years). This is similar to the study in India where 64.75% of the cases were amongst children between the ages of 0 and 6 years, 9.16% in children between the ages of 6 to 12 years, 14.30% in those between 12 years and 16 years, and 11.78% in those between the ages of 16 years and 18 years^13^. Our study, however, was for children 16 years and younger.

Most of the patients were in the lower socio-economic class. Our study found that 75% were in the lower socio-economic class whilst in Ile-Ife it was up to 98%.8 However, our findings are similar to studies conducted elsewhere.^19,20^ 

Furthermore, 97.6% of the poisoning was unintentional and is almost similar to the finding in Ile-Ife8 and in other places^17,21^. A study in America showed that 98.9% of poisoning cases occurred at home.^21^ Kerosene poisoning accounted for 56.6% of the poisoning, compared to 40.9% in Ile-Ife.^8^ In a study conducted in India, kerosene accounted for 27.9% of childhood poisoning^14^; in only about 12% out of the 468 children that were from household products were from kerosene in another study in India^13^. This shows the variation of source in terms of poisoning, even in the same country. The finding in this study is based on the exposure of kerosene to children in the Niger Delta than any other region in Nigeria. There is a refinery in Warri which is also the area in which crude oil is produced in Nigeria. Alcohol is the second most common source of poisoning. This is in contrast to the study in Ile-Ife where it was the fifth most common cause at 7.6%8; it accounted for 12.5% of cases in our study, in contrast to a study in Spain where it was responsible for about 5.9% of the cases^17^. The relatively high incidence in our study can be explained by the fact that alcohol is more frequently consumed in the region than in the Western region of Nigeria.^8^ In fact, the region is the home of the country’s native gin called ‘Sapele water’ (Sapele refers to the region where it is manufactured). The consumption of this native gin in social functions reaches almost 70%; moreover, alcohol is used as a solvent for roots and other concoctions used in the treatment of malaria, and other diseases, such as hepatitis. 

Drugs accounted for the third most common source of poisoning as opposed to caustic soda and traditional mixtures in Ife. In America, and Spain 66.7%17 of poisioning resulted from drugs and in Iran where it was responsible for 31.6% of cases with kerosene a close second at 31.2% of cases.^18^. It is the second most common cause for poisoning in India.^14^ The major drugs implicated in our study are benzodiazepines, ferrous sulphate, dibenclamide, methyldopa, metformine, cypron (corheptadine) as opposed to paracetamol and over the counter drugs in other countries like India.^14,18,22^ According to the American Association of Poison Control Centers, iron is the leading cause of childhood poisoning in children younger than 6 years. Thirty eight children between the ages of 9 months and 3 years died from iron poisoning in 2009.^21^ The drugs found as culprits in our study are used on a long-term bases by adults who carelessly keep the drugs in easy reach of children. It is not surprising that drugs result in poisoning within our environment because the Accident Association Index as enunciated by a study in Britain^24^, is high in Warri, as most drugs are not packaged in child resistant packs and come in transparent packs. Drugs are more likely to be stored in handbags and refrigerators as noted in our study, making them easily accessible to children. Ferrous sulphate is taken by children, believing it is chocolate. Poisoning from drugs was more common in the middle socio-economic class and least in the low income group.

The intentional poisoning cases were in the adolescent age group and mainly involved female teenagers; the reasons for intentional poisoning are due to social reasons and they occured more commonly at home. It is an established fact that adolescent suicidal tendencies are more common amongst female adolescents.^23^ The intentional poisoning cases occurred more often in the middle socio-economic class and also reveal the danger of keeping drugs within easy access of children and even adolescents. 

Only about 38.2% of the poisoning cases were found at the place of storage after the poisoning as found in our study. The most common place of storage is the refrigerator. A study in Hong Kong found that only 40% of the drugs were found at the place of storage and this is similar to our finding.^25^ The study in Ile-Ife did not find that drugs are a significant cause of poisoning in children, but Oshikoya and colleagues also found out a significant percentage especially from self-medication, a common problem in Nigeria.^26^ Caustic soda is not an important cause of poisoning because the populace is rarely involved in soap production. Crude oil is a routinely used substance for the treatment of convulsions. It is usually applied topically, but some confused mothers may force the convulsing children to ingest the substance. This is the observation from the authors of this study, although it has not been published yet. Two of our patients were poisoned from their mothers’ nail polish and even in America, poisoning from perfumes and cosmetics are also known.^15^ 

The majority of the children had mild symptoms which are similar to patients’ experience elsewhere.^27^ Only 11.8% of the cases were classified as severe. The complications were largely due to the use of kerosene and nail polish. 

Eleven out of the 156 patients died; 50% of the cases were due to native concoctions. This is similar to the Il-Ife study^8^; 50% of those with intentional poisoning died which was largely as a result of late presentation. 

## Conclusion

Poisoning remains a major cause of morbidity and mortality. Health education is necessary to reduce the incidence of childhood poisoning, but this education should be directed on the parents together with infrastructural development. Efforts should be made to reduce the exposure of children to poisonous substances such as kerosine. A law should be put in place which bans the storage of such products in containers similar for those used to store water and be kept very far from children. Drugs should be stored out of reach of children. In fact, a sudy in Brasil showed that storage of drugs lower than 150cm increase the chances of drug poisoning in children28 and we recommend that a law should be introduced to caution parents on ways of storing drugs. The establishment of Poison Control and Information Centres in each of the six geopolitical regions of Nigeria and a National Centre in Abuja should be introduced and given an easy telephone which can be memorised. These measures together with change in the orientation of the native inhabitants will drastically reduce the rate of childhood poisoning in Warri, in the Niger Delta.
